# Plasticizer Enhancement on the Miscibility and Thermomechanical Properties of Polylactic Acid-Chitin-Starch Composites

**DOI:** 10.3390/polym12010115

**Published:** 2020-01-05

**Authors:** Indra Surya, N. G. Olaiya, Samsul Rizal, Ikramullah Zein, N. A. Sri Aprilia, M. Hasan, Esam Bashir Yahya, K. K. Sadasivuni, H. P. S. Abdul Khalil

**Affiliations:** 1 Department of Chemical Engineering, Universitas Sumatera Utara, Medan 20155, Indonesia; indradanas@yahoo.com; 2 School of Industrial Technology, Universiti Sains Malaysia, Penang 11800, Malaysia; 3 Department of Mechanical Engineering, Universitas Syiah Kuala, Banda Aceh 23111, Indonesia; rizal@knt.mech.tut.ac.jp; 4 Department of Industrial Engineering, Universitas Serambi Mekkah: JI. Unmuha, Batoh, Kec.Lueng Bata, kota Banda Aceh, Aceh 23245, Indonesia; ikramullah.zein@serambimekkah.ac.id; 5 Department of Chemical Engineering, Universitas Syiah Kuala, Banda Aceh 23111, Indonesia; sriaprilia@unsyiah.ac.id; 6 Chemical Education Department, Universitas Syiah Kuala, Jln. Tgk. Daud Beureueh Darussalam Banda Aceh 23111, Indonesia; muhammadhasan.kimia@unsyiah.ac.id; 7 Department of Microbiology, Faculty of Science, Al Asmarya Islamic University, 218521 Zliten, Libya; essam912013@gmail.com; 8 Centre for Advance Materials, Qatar University, Doha P.O. Box 2713, Qatar; kishor_kumars@yahoo.com

**Keywords:** miscibility, extrusion, composite, biopolymer

## Abstract

In previous research, a polylactic chitin starch composite was prepared without the use of a solvent to enhance the miscibility. In this study, a polylactic acid (PLA) chitin starch composite was produced with chloroform as a plasticizer in the ratio 1:10. The blending of chitin and starch with PLA ranges from 2% to 8%. Tensile strength, impact, thermogravimetry analysis-Fourier-transform infrared spectroscopy (TGA)-FTIR, and differential scanning calorimetry (DSC) were used to test the thermomechanical properties. Also, the morphological properties, water absorption, and wear rate of the material was observed. The results showed that the tensile strength, yield strength, and impact strength were improved compared to the pure polylactic acid. Also, the elastic modulus of the samples increased, but were lower compared to that of the pure polylactic acid. The result of the fractured surface morphology showed good miscibility of the blending, which accounted for the good mechanical properties recorded in the study. The thermogravimetric analysis (TGA) and derivative thermogravimetric analysis DTA show a single degradation and peak respectively, which is also shown in the glass temperature measures from the DSC analysis. The water absorption test shows that the water absorption rate increases with starch content and the wear rate recorded sample A (92% P/8% C) as the highest. The high miscibility projected was achieved with no void, with the use of chloroform as a plasticizer.

## 1. Introduction

The role of natural polymers as a replacement of synthetic ones has been on the increase with the goal of achieving a sustainable environment. Synthetic polymers have excellent properties with various industrial applications. As the population grows, the challenge of land and marine pollution due to plastic waste is also on the increase [1,2]. Globally it is estimated that 6–12 million tons of plastic waste enters the ocean each year [1]. The marine waste pollution has resulted in the death of aquatic animals first as they feed on the plastic waste, and also due to the wounds from sharp objects from solid plastics [3]. The likely cause of death from marine pollution from plastics is higher than compared to that of oil spillage each year.

Several policies and logos have been developed on the proper disposal of plastic waste. Manufacturing companies have a symbol on their product to show the proper disposal of the packaging material after use. The use of these symbols by manufacturing to curb plastic waste pollution has not been as active as the the increase in plastic waste improper disposal. Two primary solutions have been identified to solve this problem [4]. First is the production of reusable plastic packaging material and second is the production of biodegradable packaging material. The latter seems to be a permanent solution to this problem.

The production of biodegradable packaging material to combat the problem of pollution has resulted in the use of biopolymers such as starch, cellulose, and chitin. These biopolymers are abundant on the earth and have been used and processed into different forms. Biopolymers in these forms have a significant setback in their mechanical properties. Among the processed form of starch (from corn) is polylactic acid.

Polylactic acid (PLA), which is one of the most promising natural polymers from a renewable source, has been previously researched for this purpose. PLA is similar in properties to most synthetic polymers used in industrial applications such as polyethene and nylon. Several researchers have worked on the polymer or its composite for packaging [5,6,7,8,9,10,11]. The focus of these studies are measures to improve on mechanical and thermal properties and biodegradation. Researchers that worked on polylactic acid to solve the problem of mechanical and thermal/biodegradation have done so either through blending and reinforcement. Some of the previous research has been on the use of chitin or starch independently [12].

Significant reports in the literature established that there is a need for the use of plasticizer to improve the miscibility of polylactic acid-starch polymer blends [13,14,15,16]. Jariyasakoolroj et al. [17] achieved an increase in the tensile and yield strength of polylactic acid-starch composite using silane coupling as a compatibilizer, Wu et al. [18] produced a low molecular weight PLA using various alcohols before mixing with starch. Good miscibility and degradation were achieved using various alcohols but with reduced mechanical properties due to reaction of the plasticizer with the composite. Also, Clasen et al. [19] used maleic anhydride as a plasticizer. An improvement was noticed in the miscibility of the resulting composite, but the mechanical properties were affected. Kasinee et al. [20] use poly(methyl methacrylate) to modify starch before mixing with PLA. The resulting polymer composite was hydrophobic, and the tensile and yield strength was improved.

Also, previous work on polylactic acid–chitin composites resulted in similar challenge of immiscibility. According to reported literature, use of a plasticizer was suggested by some researchers. As an example, Grande et al. [21] worked on improving the miscibility of polylactic acid-chitin by using polyvinyl alcohol and glycerol as compatibilizer and plasticizer respectively. They reported that chitosan spread in the composite as particles in the melt blend. Maria et al. [22] in their work on plasticized polylactic acid using tributyl o-acetyl citrate (ATBC) mixed with chitosan. Good mixability was achieved with improved mechanical and antimicrobial activities. Fathima et al. [23] used polyethene glycol as a cross-linking agent and polyvinyl alcohol as a plasticizer to produce a polylactic acid chitin composite with improved mechanical properties.

All the studies focused on the use of starch or chitin at different time. Starch is found to improve the degradation properties of PLA and chitin the mechanical properties, but the effect of starch–chitin blend on PLA has not been researched; hence, the novelty of this work. Also, the use of chloroform as the medium of miscibility for the three polymers has not been researched. This study seeks to produce a polylactic-chitin-starch with improved miscibility using chloroform. The mechanical properties, thermal degradation, and morphology were investigated.

## 2. Materials and Methods

### 2.1. Materials

Pelletized polylactic acid 4044D reactive extrusion grade was used for the experiment. The PLA, acquired from NatureWorks (Minnetonka, MN, USA), with a specific gravity of 1.24, glass temperature between 55–60 °C, mass flow rate (MFR) (g/10 min), relative viscosity 4.0, semi-crystalline with melting peak between 150–170 °C, and solubility of 1.0 g/dL in chloroform at 30 °C. The tensile strength and modulus are 58 MPa and 3387 MPa respectively. Corn starch (particle size range 0.050 µm to 1 µm) and practical grade chitin (shrimp, particle size range 0.050 µm to 1 µm) from Sigma Aldrich (Selangor, Malaysia) in powdered form were used for the experiment. Chloroform was purchased from ChemPur (Selangor, Malaysia).

### 2.2. Composite Preparation

The polylactic acid (P) was kept at a constant percentage (92 wt %) while the starch (S) varies from 2% to 8% and chitin (C) also varies from 2% to 8% of the composition. Five samples were produced A, B, C, D, and E with blend percentage variation in chitin (C) and starch (S); P8C, P6C2S, P4C4S, P2C6S, and P8S respectively. The 100% polylactic acid is labelled sample F.

The polylactic pellets were dried in a vacuum dryer (drying ovens, sterilizers UM series, Lilienthal, Germany) for 24 h at 60 °C. The PLA pellets were dissolved in chloroform (CF) and mechanically stirred until the pellets were dissolved at 60 °C. The PLA/CF were mixed in the ratio 1:10, and mechanical stirring was applied for 6 h to form a uniform mix of the blend. Starch and chitin were added to the mixed PLA/CF. The mix was then extruded using a twin-screw extruder (LABTECH, Samutprakarn, Thailand) into filament. The filament was later pelletized using LABTECH pelletizer (LABTECH, Samutprakarn, Thailand). The pellets were air-dried for 24 h at 40 °C using in Memmert drying cabinet model ULM 500 (drying ovens, sterilizers UM series, Lilienthal, Germany). The composite pellets were moulded into test samples at 150 °C using a Carver press compression (Carver 2697 Hydraulic Heated Platen Press Bench Mod, Warwickshire, UK) moulding machine. The samples were stored in zip-lock bags

### 2.3. Characterization

#### 2.3.1. Tensile Test

The tensile test was done using a MT1175 (Dia-Stron Instruments, Andover, UK) at ASTM D3039 for the polymer composite. Standard test samples were prepared, and the results for the tensile strength, tensile modulus and yield strength were obtained.

#### 2.3.2. Impact test

The impact test was done using a Ceast Resil 7181 Impactor (Corporate Consulting, Service and Instruments (CCSi), Akron, OH, USA) to obtain the resilience of the material in Joules per square meter. A standard test D256 was used for the test with specified dimensions. The composite morphological properties were done using scanning electron microscopy (EVO MA 10, Carl-ZEISS SMT, Oberkochen, Germany). The fractured surface morphology of the impact samples was done to examine the miscibility of the blend using plasticizer.

#### 2.3.3. Thermogravimetry analysis (TGA)–FTIR Test

The thermogravimetry analysis (TGA-DTA) was conducted using a Mettler-Toledo thermogravimetric analyzer model TGA/DSC 1, (Mettler-Toledo, Greifensee, Switzerland). A mass range of 20 mg to 22 mg was used for the test at a temperature range of 20 °C to 900 °C. The weight loss and derivative weight loss against temperature values was obtained for the TGA and DTA respectively. The test was done in an air environment. Also, the FTIR analysis (Perkin-Elmer, PC1600, Winter Street Waltham, MA, USA) was done as a standard test. The analysis procedure was done with an absorption spectrum range of 400 to 6000 cm^−1^.

#### 2.3.4. Differential Scanning Calorimetry Analysis

The differential scanning calorimetry was done, using Perkin–Elmer differential scanning calorimetry (DSC) model 6 (Perkin–Elmer, Schwerzenbach, Switzerland), to further analyses the behaviour of the material with temperature change. Measurement of the glass transition temperature, crystallization, and melting temperatures were recorded with a mass range between 5 mg to 7 mg.

#### 2.3.5. Abrasive Wear Test

The wear rate of the composite was done to estimate and study the wear behaviour of the materials using a ball-on-disk tribological technique. ASTM G99-05 standard test method was used to carry out the dry sliding wear tests with a ball-on-disk tribometer (UMT-2, Bruker, formerly known as CETR, Bruker, Billerica, MA, USA). Continuous monitoring of the coefficient of friction (COF) was observed throughout the test; a mean COF was determined and reported. The wear volume was determined by continuously recording the weight difference before and after each test using a very sensitive analytical weighing balance (Shimadzu AY120) which can weigh up to 0.0001 g. The volume material loss (V) of the samples was calculated using the equation

V = ∆w/ρ(1)

where w = weigh before test–weight after test and ρ = density of the various nanocomposites. Finally, the specific wear rate, k, was determined based on the Lancaster relationship

k = V/F_N.(2)

#### 2.3.6. Water Absorption Test

The water absorption rate was determined by measuring the initial mass and the final mass of the samples after 72 h of immersion in water. The samples were oven-dried, and the water absorption was determined using American Society for Testing and Materials (ASTM) test method D570-81. The contact angle was also determined using the mean value of the 5 mL water released to the surface of the composite and shot with a contact angle analyzer (KSV CAM 101; KSV Instruments Ltd., Helsinki, Finland).

## 3. Results

### 3.1. Tensile Properties

**Figure 1:**
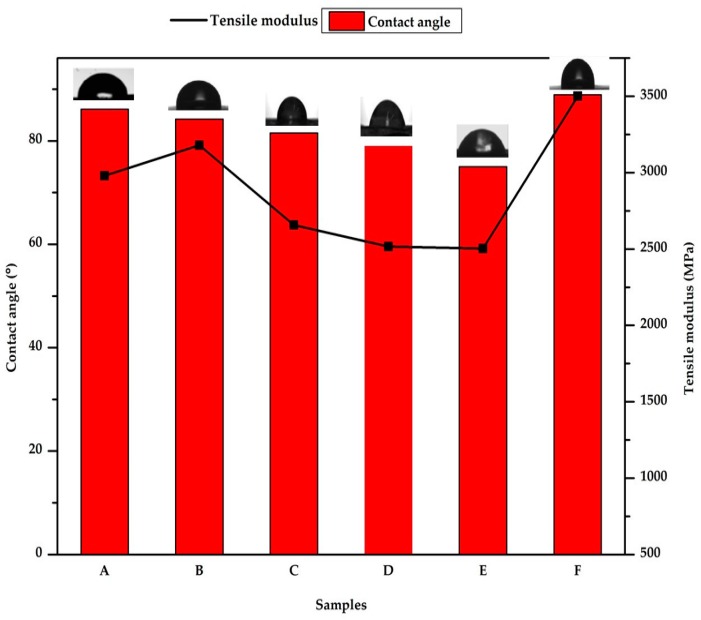
Tensile modulus and contact angle of samples A—92% polylactic acid (P)/8% chitin (C); B—92% P/6% C/2% starch (S); C—92% P/4% C/4% S; D—92% P/2% C/6% S; E—92% P/8% S; F—100% P.

**Figure 2:**
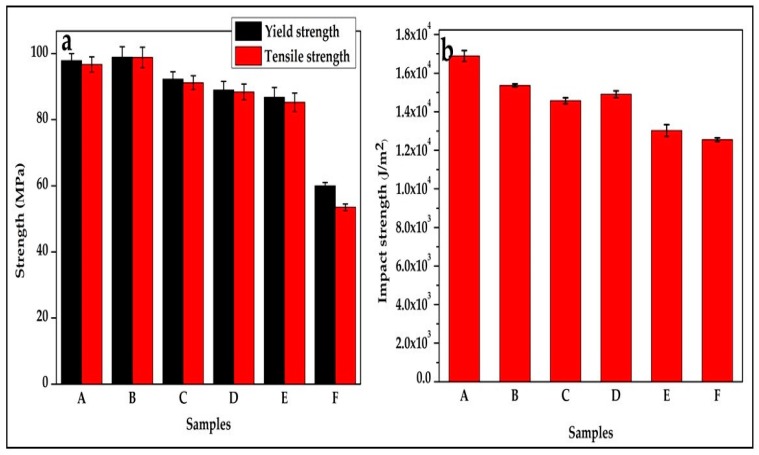
(**a**) Tensile properties and (**b**) Impact strength of samples A—92% polylactic acid (P)/8% chitin (C); B—92% P/6% C/2% starch (S); C—92% P/4% C/4% S; D—92% P/2% C/6% S; E—92% P/8% S; F—100% P.

The tensile, yield, and tensile modulus result of plasticized polylactic acid composite for the six samples is shown in Figure 1 and Figure 2. The result shows an increase in the values of the tensile strength when compared with that of pure PLA (sample F). The tensile strength is seen to reduce with an increase in starch content and decrease chitin content. This is an indication of the miscibility of the three polymer blends [12,24]. Also, the increase in tensile strength is between 59% to 85%. The tensile strength is seen to have the highest value at 6% of chitin and 2% of starch (sample B) blending. This result is in correlation with the tensile properties reported by [25]. In their report, the tensile strength of the PLA composite increase with chitin content from 1% to 5% blending, but a drop is noticed in the composite with 10% reinforcement. Also, in the work of [26] on PLA-starch, an increase in tensile strength was reported with reduced starch content.

The yield strength was improved, which can be attributed to the effect of the plasticizer on the blend. Sample B was seen to have the highest yield strength. Comparing with the literature of previously produced polylactic acid starch and polylactic chitin, the report of [24] on polylactic chitin with glycerol as plasticizer showed a similar trend in yield strength.

The tensile modulus value reduced compared with the pure polylactic acid [27], but the value of the modulus increases with an increase in chitin percentage and reduction in starch. Sample B was seen to have the highest yield strength and young modulus value. Comparing with the literature of previously produced polylactic acid starch composite and polylactic chitin composite, the report of Nasrin et al. [24] on polylactic chitin with glycerol as plasticizer showed a similar trend in yield strength and elastic modulus. The excellent mechanical properties of the composite are probably due to the combined effect of the physical mobility of the constituent, enabled using chloroform and the compatibility interfacial bonding by the chitin [12,25].

The contact angle, which describes the wettability of the polymer composite, is seen to decrease with an increase in the percentage of starch and is generally lower than 90 for the composite samples. The neat PLA is seen to have the highest contact angle. The polysaccharides (chitin and starch) are seen to improve the wettability of the neat PLA. The contact angle of PLA/chitin is next in value to neat PLA and the contact angle drop with the addition of starch content. The lowest is at 8% starch (sample E, Figure 1).

### 3.2. Impact and Morphological Properties

The impact strength of the resulting composite is shown in Figure 2. The impact strength is higher compared with the neat polylactic acid (sample F). Sample A is seen to have the highest impact strength, and this may be due to the presence of a high percentage of chitin in the blending. The impact strength, when blended with chitin or starch, has been reported to increase more than pure PLA [28]. The impact strength is lowered with increased content of starch in the composite because the effect of chitin content on the impact strength is high. Also, the interfacial adhesion of PLA and chitin plays a significant factor in the value of the impact strength. The interfacial adhesion between PLA and starch is seen to reduce with increased starch content as reported by [27].

**Figure 3:**
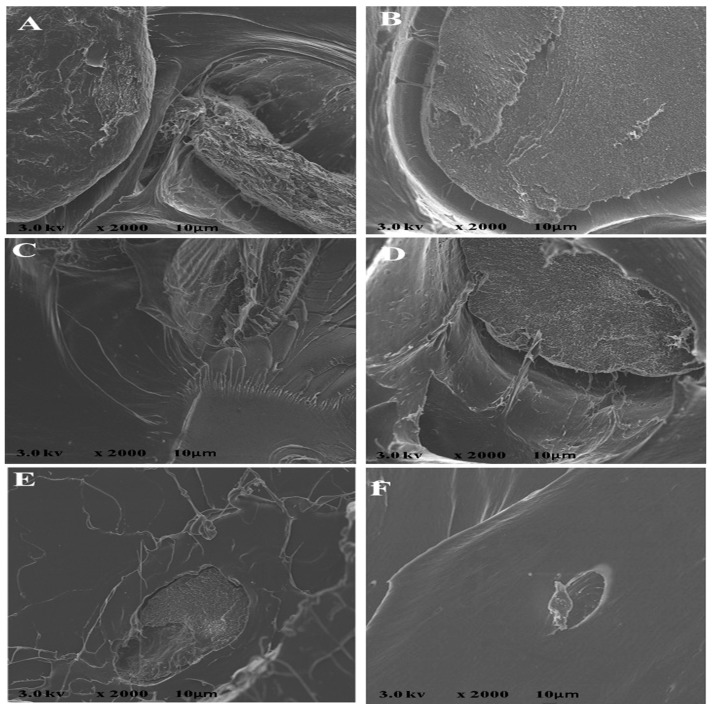
Scanning electron microscope images for samples with the ratios A—92% polylactic acid (P)/8% chitin (C); B—92% P/6% C/2% starch (S); C— 92% P/4% C/4% S; D—92% P/2% C/6% S; E—92% P/8% S; F—100% P.

The scan electron microscopy of the impact fractured surface shows a smooth morphology of the fractured samples (Figure 3). There is high miscibility of the polymer blend and this accounts for the good mechanical strength of the composite. This miscibility of the polymer composite can be attributed to the use of chloroform as the plasticizer and the interfacial bonding of PLA/chitin and chitin /starch. Chitin has been reported to act as a compatibilizer between PLA and starch [12,24,25,29,30]. The images are characterized with network flakes of starch–chitin blends and edges with no voids. The researchers in [31] reported the same experience using glycerol/sorbitol as a plasticizer for polylactic starch. The report stated that fine microstructure was as a result of the use of a plasticizer. This was also accompanied by improvement in mechanical properties, but low crystallization, as seen in this study.

### 3.3. Thermogravimetry Analysis Result

**Figure 4:**
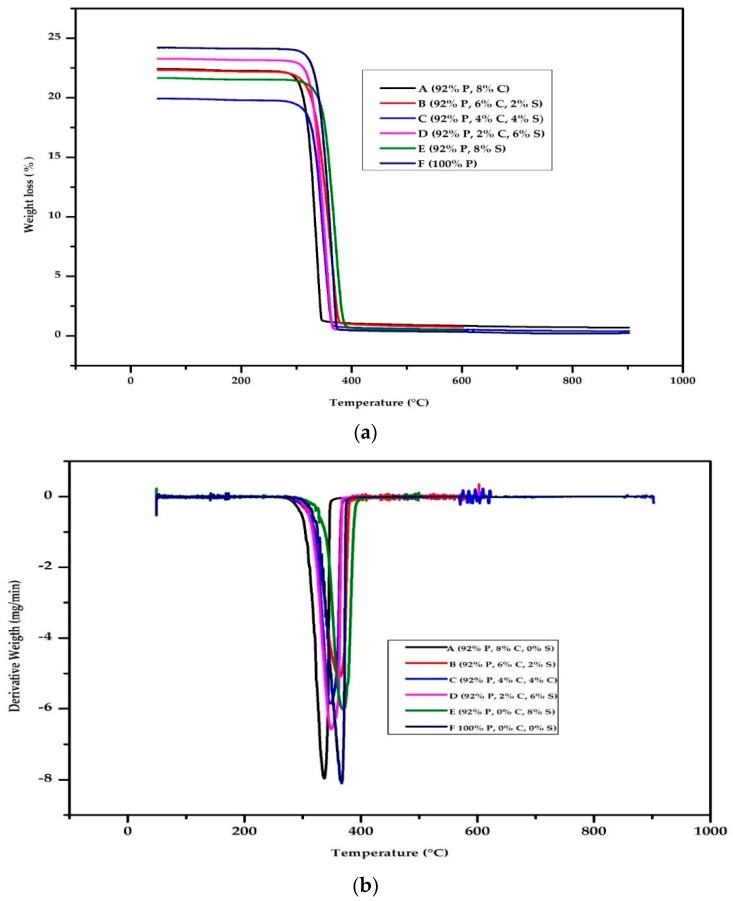
(**a**) Thermogravimetry analysis (TGA) result of samples A—92% polylactic acid (P)/8% chitin (C); B—92% P/6% C/2% starch (S); C—92% P/4% C/4% S; D—92% P/2% C/6% S; E—92% P/8% S; F—100% P. (**b**) Derivative thermogravimetric analysis (DTA) result of samples A—92% polylactic acid (P)/8% chitin (C); B—92% P/6% C/2% starch (S); C—92% P/4% C/4% S; D—92% P/2% C/6% S; E—92% P/8% S; F—100% P.

The result of the TGA - DTA, is shown in Figure 4a,b. The TGA curve (Figure 4a) shows a single drop, with no initial weight loss, in its decomposition curve, and this is also verified from the DTA curve. The weight loss for samples A, B, C, D, and E started at 254 °C, 253 °C, 278 °C, 249 °C, and 247 °C respectively with maximum weight loss at 345 °C, 385 °C, 379 °C, 379 °C, and 385 °C. The lowered value of the onset temperature decreases with increase in starch content, and this has been reported by [18,26]. The thermal degradation behaviour of sample A and B look similar which shows domination of the chitin content, but above 312 °C, during the degradation, a slit difference was noticed.

The drop-in weight loss of samples A, B, C, D, and E is 97%, 97%, 96%, 94%, and 98%. This shows that samples decomposition percentage drop is high with less than 6% of residue. Sample E having the highest decomposition percentage can be traced to the characteristic of high starch content. Starch has been reported to improve the degradation behaviour of PLA [26,27]. Generally, the degradation is improved compared with pure PLA (sample F) and this could be as a result of starch or plasticizer, as reported by [27]. Also, the TGA curve shows the presence of residue after the decomposition region as a continuous horizontal line which is expected from the combustion of polymers.

The DTA curve (Figure 4b) shows an endothermic peak with values for samples A, B, C, D, and E at 336 °C, 357 °C, 364 °C 348 °C, and 369 °C respectively and this shows the different application where the material can be used. Above this temperature, the material fails. The curve also reveals that sample E has the highest decomposition temperature, which is typical of the presence of starch.

### 3.4. FTIR Properties

**Figure 5:**
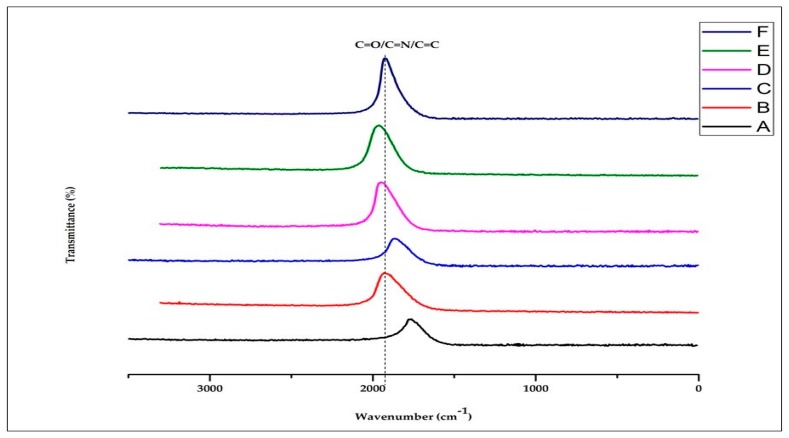
FTIR plot of samples A—92% polylactic acid (P)/8% chitin (C); B—92% P/6% C/2% starch (S); C—92% P/4% C/4% S; D—92% P/2% C/6% S; E—92% P/8% S; F—100% P.

**Figure 6:**
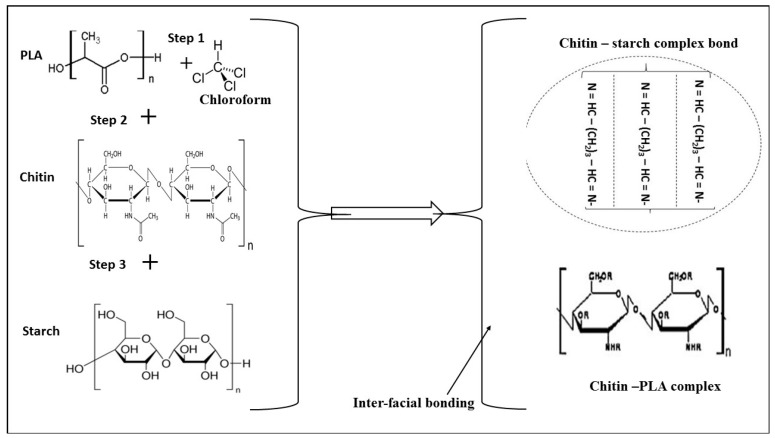
Picture representation of the interfacial bonding between polylactic acid (PLA)–chitin and chitin–starch.

The FTIR result (Figure 5) shows the possible bond present during the decomposition and the gas involved during the thermal analysis. It also reveals the possibility of present residue. The FTIR result shows possible bonds of C=O, C=N, and C=C for wavenumber 1500 cm^−1^ to 2000 cm^−1^. The C=O and C=N are most likely from the chitin content. Also, the plasticizer seems not to be reflected in the FTIR result and no –CH_3_ or C–Cl bond is noticed in the result. The C=O and C–H are generally expected from polymers, but the C–N most likely is from the chitin decomposition. The formation of interfacial bonding is shown in Figure 6.

### 3.5. Differential Scanning Calorimetry

**Figure 7:**
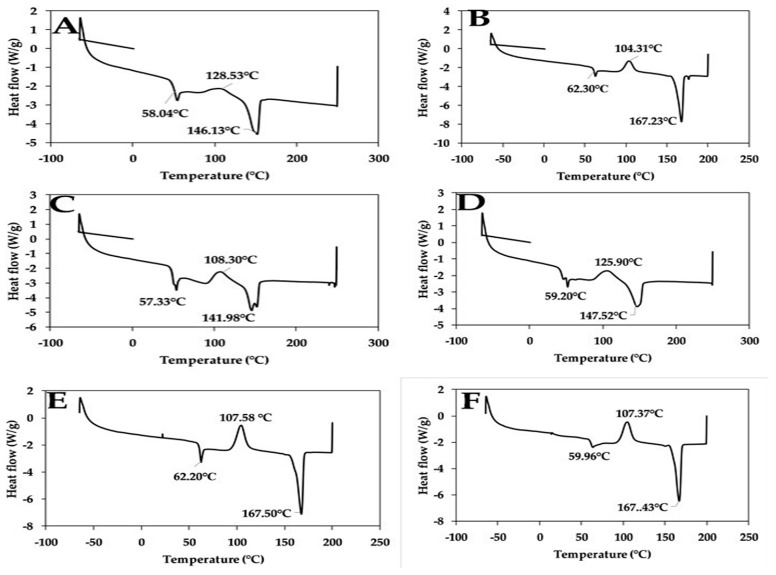
DSC plot of samples A—92% polylactic acid (P)/8% chitin (C); B—92% P/6% C/2% starch (S); C— 92% P/4% C/4% S; D—92% P/2% C/6% S; E—92% P/8% S; F—100% P.

Figure 7 shows the graphical representation and Table 1 shows a summary of the results of the differential scanning calorimetry. The table shows the glass temperature, melting temperature and crystalline temperature of the samples. Also, the heat of melting and crystallization is reported in the table. The DSC cure shows single sharp peaks for the glass temperature, which shows the compatibility of the polymer using the plasticizer. The glass transition temperature is seen to be highest with sample E composite and lowest with sample C, likely due to the difference in the nature of chitin and starch, which are in equal proportion. The peak and crystallization temperatures of the samples are seen to increase with the increase in starch content and reduced chitin content. Samples C and D are seen to have low enthalpy at crystallization and melting, which shows the composite cannot retain heat. A single endothermic peak was observed for the melting against the usual double peak for the PLA composite reported by [27].

### 3.6. Wear Properties

**Figure 8:**
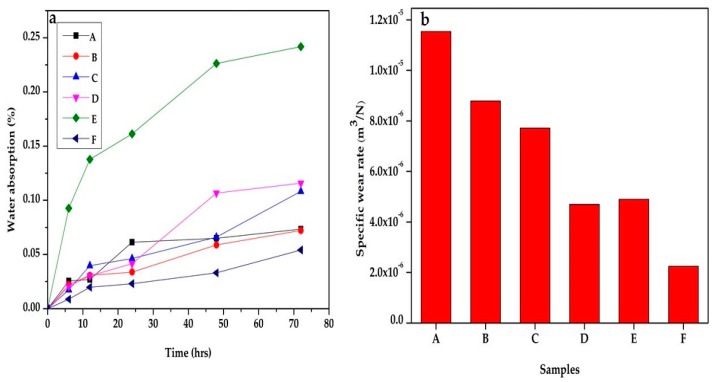
(**a**) Wear and (**b**) water absorption properties of samples A—92% polylactic acid (P)/8% chitin (C); B—92% P/6% C/2% starch (S); C— 92% P/4% C/4% S; D—92% P/2% C/6% S; E—92% P/8% S; F—100% P.

The wear test was done to study the response of the material to abrasive force to know the applicability of the material to a specific application. The wear properties of the polymer composite state its response to rubbing of scrubbing when in use. In this study, the effect of the blending of the polysaccharides (chitin and starch) on the wear properties of PLA is investigated (Figure 8a). Generally, the wear resistance of the composite is reduced compared to the neat PLA (sample F). The wear resistance of the composite increases with an increase in starch content and decreasing chitin content. The explanation for this is that as the starch content increases, the friction on the surface of the polymer composite reduces. Therefore, the wear rate reduces. This is logical as the abrasive wear is dependent on the surface friction. A similar result was reported by [32] in their research on PLA/starch composites.

### 3.7. Water Absorption

The water absorption is an essential property of a biodegradable polymer composite. The water absorption of the composite in this study increased with the percentage of starch and decreasing chitin content. The effect of starch content is more significant than that of chitin, from Figure 8b. The starch in the polymer composite increases the water absorption due to the formation of hydrogen bonding between the water molecule and the starch. Sample E (with the highest starch content) has the maximum water absorption, and the neat PLA has the least. The water absorption of starch is attributed to the hygroscopic nature of the polysaccharide. This result is similar to those in [33,34,35,36,37].

## 4. Conclusions

The thermochemical properties of polylactic acid blends with chitin and starch were studied. The results show good mechanical properties in terms of tensile strength, yield, and impact, with sample B having the highest value. The thermogravimetry analysis shows that the composites are stable thermally with a single degradation curve and glass transition temperature. The morphological properties show good compatibility and fine structured network distribution of the blends with no void. The composite is a good substitute for synthetic composites for plastic packaging based on the results.

## Figures and Tables

**Table 1:** Summary of the results for differential scanning calorimetry (DSC).

Samples	Mass	Glass Temp.	Crystallization			Melting		
	(g)	°C	Peak Temp. °C	Crys. Temp. °C	Heat of Crys. J/g	Peak Temp. °C	Melting Temp. °C	Heat of Melting J/g
A	5.67	58.04	128.53	113.55	1.77	146.13	141.35	1.56
B	6.80	62.30	104.31	96.37	30.45	167.23	161.73	38.69
C	6.63	57.33	108.30	99.65	4.209	141.98	131.25	4.368
D	5.51	59.20	125.90	109.77	6.233	147.52	141.86	7.761
E	5.70	62.20	107.58	98.07	28.06	167.5	162.17	36.41
F	5.60	59.96	107.37	97.56	27.49	167.43	162.12	32.32

Temp—Temperature; Crys.—Crystallization.
